# Effects of responsive caregiving and learning opportunities during pre-school ages on the association of early adversities and adolescent human capital: an analysis of birth cohorts in two middle-income countries

**DOI:** 10.1016/S2352-4642(20)30309-6

**Published:** 2021-01

**Authors:** Angela C B Trude, Linda M Richter, Jere R Behrman, Aryeh D Stein, Ana M B Menezes, Maureen M Black, 1993 Pelotas and Birth to Twenty Plus investigators

**Affiliations:** a Growth and Nutrition Division, Department of Pediatrics, University of Maryland School of Medicine, Baltimore, MD, USA; b DSI-NRF Centre of Excellence in Human Development, University of the Witwatersrand, Johannesburg, South Africa; c Economics Department, The Ronald O Perelman Center for Political Science and Economics, University of Pennsylvania, Philadelphia, PA, USA; d Hubert Department of Global Health, Rollins School of Public Health Emory University, Atlanta, GA, USA; e Postgraduate Program of Epidemiology, Federal University of Pelotas, Pelotas, Brazil; f RTI International, Durham, NC, USA

## Abstract

**Background:**

Millions of children globally are at risk of not reaching their developmental potential because of early adversities. We hypothesised that responsive caregiving and learning opportunities, components of nurturing care, at pre-school ages might mitigate the effects of adversities.

**Methods:**

We analysed longitudinal birth cohort data from Brazil (1993 Pelotas Birth Cohort, n=632) and South Africa (Birth to Twenty Plus [Bt20+] Birth Cohort, n=1130) to assess whether responsive caregiving and learning opportunities at pre-school ages (2–4 years) modified associations between cumulative early adversities and adolescent human capital. The cumulative adversities score (range 0–9) included household wealth and crowding; mothers' schooling, height, age, and mental health; and children's birthweight, gestational age, and length at age 12 months. We extracted data on responsive caregiving and learning opportunities from the Early Childhood Home Observation for Measurement of the Environment inventory, assessed at age 4 years (1993 Pelotas cohort) and 2 years (Bt20+ cohort). We examined three human capital indicators: intelligence quotient (IQ) assessed at age 18 years (1993 Pelotas cohort) and 16 years (Bt20+ cohort); psychosocial adjustment assessed at age 15 years and 14 years, respectively; and height assessed at age 18 years and 16 years, respectively. We used linear models with interaction terms between cumulative adversities, and responsive caregiving and learning opportunities, to predict adolescent human capital.

**Findings:**

For each additional Z score of total cumulative adversity, adolescent IQ decreased by 5·89 (95% CI −7·29 to −4·50) points in the 1993 Pelotas cohort (p<0·0001) and 2·69 (–4·52 to −0·86) points in the Bt20+ cohort (p=0·0039). After adjusting for total cumulative adversities, adolescent IQ points increased by 5·47 (95% CI 4·20 to 6·74) with each additional Z score of learning opportunities and by 2·26 (0·93 to 3·59) with each additional Z score of responsive caregiving in the 1993 Pelotas cohort, but not in the Bt20+ cohort (0·86 [–0·12 to 1·83] and 0·65 [–0·32 to 1·61], respectively). Associations between early adversities and IQ were modified by learning opportunities in the 1993 Pelotas cohort (beta coefficient for interaction 1·74, 95% CI 0·43 to 3·04; p=0·0092) and by responsive caregiving in the Bt20+ cohort (2·24, 0·94 to 3·54; p=0·0075). High nurturing environment attenuated the negative effects of early cumulative adversities on IQ.

**Interpretation:**

Early nurturing home environments protect young children against effects of early adversities on adolescent IQ, with long-term positive associations on adolescent cognition in two middle-income countries.

**Funding:**

Bill & Melinda Gates Foundation.

## Introduction

Many children in low-income and middle-income countries (LMICs) are at risk of not reaching their developmental potential,^1^ mainly due to early adversities, including poverty, poor health and nutrition, and insufficient protection, responsive care, and learning opportunities. In LMICs, more than 40% of children (≤18 years) live in extreme poverty (<US$1·90 per day purchasing power parity exchange rate), which increases their likelihood of experiencing multiple adversities,^2^ undermining cognition, psychosocial adjustment, and health.^3^ Early adversities not only affect individuals' health and wellbeing throughout the life course, but also result in poor returns to society, including loss of abilities, knowledge, and social skills required for producing economic value and fostering human capital.^4^ In addition, the accumulation of multiple adverse experiences is associated with worse health and development than single risk factors, attributed to the complex interplay of co-occurring and overlapping risks disproportionally associated with poverty.^5^ Building on the bioecological theory of human development,^6^ children might cope with single risks, but cumulative risks often break down coping strategies.^3^

**Research in context**

**Evidence before this study**

Globally, more than 250 million children younger than 5 years are at risk of not reaching their developmental potential, leading to loss of human capital with long-term effects on individuals and societies. Adversities that co-occur early in life and accumulate with age, including extreme poverty, and poor health and nutrition, undermine children's cognitive, psychosocial, and physical development. Evidence suggests that the negative effects of early cumulative adversities on children's development can be modified by nurturing care. We searched PubMed and PsycInfo for systematic reviews and meta-analyses published between Jan 1, 2010, and Dec 31, 2019, using the terms “child development” and “nurturing care”, “nurturing care framework”, “early stimulation”, “early learning”, or “responsive caregiving” and “human capital” and “cumulative risk” or “cumulative adversity”. We also searched for longitudinal studies and randomised controlled trials testing moderation effects of nurturing care on child and adolescent human capital in low-income and middle-income countries. Programmes that promote learning and responsive caregiving, components of nurturing care, can enhance children's development. However, little is known about their effects on development into adolescence, especially in low-income and middle-income countries, and whether a nurturing home environment protects young children from the negative consequences of early cumulative adversities.

**Added value of this study**

Our findings add a longitudinal component (up to adolescence) to previous studies showing that responsive caregiving and learning opportunities can buffer against individual, family, and community threats, and promote child development. We used prospective data on early cumulative adversities, components of home nurturing care, and proxies of human capital measured at similar ages in two large, longitudinal, population-based cohorts in South Africa and Brazil, strengthening the conceptual model linking nurturing care to adolescent human capital. Our findings were consistent with cumulative adversity research, with higher early cumulative adversities having inverse associations with human capital in both settings. Nurturing care in the home during the preschool years was positively associated with adolescent human capital. Our moderation models showed that the negative effects of cumulative adversities on cognitive development (intelligence quotient; IQ) were mitigated by learning opportunities in Brazil. In both sites, among youth with high learning opportunities and responsive caregiving at age 4 years, there were no significant effects of cumulative adversities on adolescent IQ. We did not find evidence that either responsive caregiving or learning opportunities mitigated the effects of early cumulative adversities on adolescent psychosocial adjustments or height.

**Implications of all the available evidence**

Children with multiple early cumulative adversities are at risk of poor human capital development. Responsive caregiving and learning opportunities can mitigate the negative consequences of adversities on cognitive development. Ensuring that all children have opportunities to realise their developmental potential enhances human capital development, facilitates attainment of the Sustainable Developmental Goals, and reduces inequities. Implementing policies and programmes that enhance nurturing care among families and communities in which children are susceptible to early cumulative adversities can possibly promote human capital development.

The negative effects of early adversities vary across children, in response to modifiable environmental factors. Globally, home environments characterised by responsive caregiving and learning opportunities have been positively associated with child development.^7^ Parenting interventions and services in LMICs have incorporated responsive caregiving and learning opportunities, with positive effects on children's attachment, cognition, and psychosocial development.^7^, ^8^ As proposed in the Nurturing Care Framework,^2^ children need responsive caregiving and learning opportunities to develop, in addition to health, nutrition, and security and safety.^8^

Modifying home environments has been effective in altering development among children with stunting. For example, a Jamaican home-visiting programme that improved learning opportunities for children with stunting resulted in significant gains in human capital, measured by intelligence and school performance in childhood and adolescence, and prosocial behaviours and earnings in adulthood.^9^ In India, nurturing home environments mitigated negative associations between stunting, and child motor and language development.^10^

**Figure 1:**
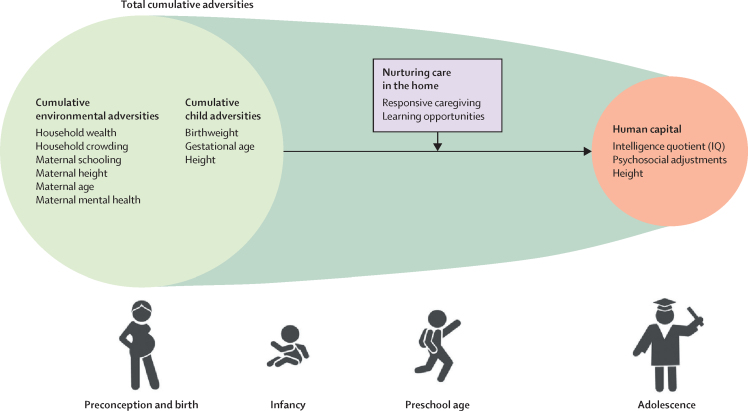
Conceptual model of the proposed association between early cumulative adversities and aspects of human capital in adolescence that might be modified by nurturing care in the home Nurturing care in the home and adolescent human capital are embedded within cumulative adversities throughout the life course.

Our premise is that nurturing care can alter the course of development, extending into adolescence, among children exposed to cumulative adversities in LMICs. Using data from large longitudinal birth cohorts in two different sociocultural contexts, we tested the hypotheses that home-based responsive caregiving and learning opportunities (nurturing care components) during pre-school ages (2–4 years) are positively associated with intelligence quotient (IQ), psychosocial adjustments, and height in adolescence, and that these opportunities mitigate the inverse associations between cumulative adversities and adolescent human capital (figure 1).

## Methods

### Study design

We used longitudinal data from early childhood into adolescence from ongoing prospective birth-cohort studies in Brazil (1993 Pelotas Birth Cohort) and South Africa (Soweto, Johannesburg, Birth to Twenty Plus [Bt20+] Birth Cohort).

The 1993 Pelotas Birth Cohort is an ongoing population-based study designed to evaluate health and development across the lifespan. Pelotas is in the south of Brazil, and has a population of approximately 350 000 inhabitants.^11^ Children from the 1993 Pelotas cohort grew up when the country was transitioning from primarily rural low income, to primarily urban middle income with a unified health-care system and, starting in 2001, a large conditional cash transfer programme.^12^ At the beginning of the study, the mortality rate was 21 per 1000 births.^13^ 5249 of the 5265 babies born in 1993 in Pelotas were enrolled in the birth cohort.^14^ Some visits and measures were done in a random subsample for logistical and financial purposes.^14^ The cohort assessment done at age 18 years had a follow-up rate of 81·4%.^15^ Each assessment was approved by the Research Ethics Committee of the Federal University of Pelotas School of Medicine. Participants (and their mothers at the early ages) provided written informed consent at each stage of the study.

Bt20+ is an ongoing longitudinal birth cohort study in the metropolitan area of Soweto, South Africa,^16^ with the overall aim to evaluate health and wellbeing of children growing up in a rapidly urbanising environment.^17^ Soweto is a township adjacent to the city of Johannesburg in the province of Gauteng, with an estimated population of more than 2·5 million people. The Bt20+ cohort represents the first generation of children born in a democratic system after the breakdown of the apartheid state. At the national level, there has been increased urbanisation; improved access to child and health-care services and technology; and reduced poverty after the expansion of several remedial social programmes.^18^ Inequality and poverty remain high, with 20% of the Soweto population currently living in households with income below the poverty line (based on the cost of a reference food basket, approximately US$47 per month).^19^ Enrolment into Bt20+ started during pregnancy (gestational age between 26 weeks and 40 weeks) among women living in Soweto in 1990. Eligible babies and their caregivers were enrolled in the study (3273 dyads) and have been followed up frequently. The assessment done when participants were aged 17 years had a follow-up rate of 70·6%.^16^ Ethical approval for the study was granted by the Committee for Research on Human Subjects at the University of Witwatersrand, South Africa, and consent was obtained from all participating women, with assent or consent also obtained from children and adolescents.

### Procedures

We examined the modifying effects of nurturing care in the home on the association between cumulative adversities and adolescent human capital (panel).

Panel **Overview of main measures used in the analyses and years of assessment across the two birth cohorts**

**Outcome (human capital)**

*1993 Pelotas Birth Cohort*

• Intelligence quotient (IQ): Wechsler Adult Intelligence Scale (WAIS-III short form; 18 years)
• Psychosocial adjustments: Strengths and Difficulty Questionnaire (15 years)
• Height: height-for-age Z score (18 years)

*Birth to Twenty Plus (Bt20+) Birth Cohort*

• IQ: Raven's Standard Progressive Matrices (16 years)
• Psychosocial adjustments: Youth Self-Report (14 years)
• Height: height-for-age Z score (16 years)

**Effect modifier (nurturing care in the home)**

*1993 Pelotas Birth Cohort*

• Responsive caregiving: responsiveness of Early Childhood Home Observation for Measurement of the Environment (EC-HOME; seven questions, numbers 26–32; 4 years)
• Opportunities for learning: Learning Materials and Language Stimulation of EC-HOME (18 questions; 4 years)

*Bt20+ Birth Cohort*

• Responsive caregiving: six questions based on EC-HOME (2 years)
• Opportunities for learning: five questions based on EC-HOME (2 years)

**Cumulative adversities**

*1993 Pelotas Birth Cohort*

• Household wealth: self-report income (birth)
• Maternal schooling: self-report grades attained (birth)
• Maternal height: measured in cm (birth)
• Maternal age: self-report (birth)
• Maternal mental health when child was young: self-report questionnaire (4 years)
• Household crowding: total people per room (4 years)
• Birthweight: measured in grams (birth)
• Gestational age: self-report from day of last period (birth)
• Length at 12 months: length, length-for-age Z score (12 months)

*Bt20+ Birth Cohort*

• Household wealth: self-report asset index (birth to 2 years)
• Maternal schooling: self-report grades attained (antenatal to 2 years)
• Maternal height: measured in cm (antenatal to 2 years)
• Maternal age: self-report (birth to 2 years)
• Maternal mental health when child was young: Pitt Depression Inventory (6 months)
• Household crowding: total people per room (antenatal to 2 years)
• Birthweight: measured in grams (birth)
• Gestational age: self-report from day of last period (birth)
• Length at 12 months: length, length-for-age Z score (12 months)

Human capital is defined as the education, training, skills, and health that contribute to economic and other forms of productivity and social integration.^20^ We included the domains of adolescent IQ, psychosocial adjustment, and height assessed with differing, but conceptually equivalent, methods. In the 1993 Pelotas cohort, IQ at age 18 years was assessed using four subtests of the Wechsler Adult Intelligence Scale (WAIS-III short form; n=4050), namely similarities, picture completion, arithmetic, and symbol coding. The WAIS-III score was adapted and standardised for Brazil and normalised for analysis. In the Bt20+ cohort, cognitive development was assessed with the Raven's Standard Progressive Matrices test at age 16 years (n=1373). This test measures non-verbal cognitive functioning and has been used widely, including in South Africa.^21^ We measured psychosocial adjustment with reverse scaling of internalising and externalising problems.^22^ In the 1993 Pelotas cohort, psychosocial problems were assessed at age 15 years with the Strengths and Difficulty Questionnaire (SDQ) parent version, a brief screening tool adapted and validated for Brazil.^23^ Mothers answered 20 questions representing internalising and externalising items about their adolescents' behaviours. Parental responses are valid for assessing adolescents' internalising and externalising behaviours.^24^ In the Bt20+ cohort, the 112-item Youth Self-Report^25^ was completed by adolescents at age 14 years. Behaviours were rated on a 3-point scale (from not true [score 1] to often true [score 3]). For this analysis, we used the 12-item anxiety (internalising) and 14-item aggression–oppositionality (externalising) items that are most comparable with the SDQ items. For both cohorts, the questionnaires referenced the preceding 6 months. We summed the reverse-scaled items to generate scores denoting positive psychosocial adjustment, consistent with the direction of the other two measures of human capital. Standing heights were assessed using stadiometers in the 1993 Pelotas cohort at age 18 years, and at age 16 years in the Bt20+ cohort, matching the ages of IQ measurement. Heights were converted to Z scores using age-specific and sex-specific WHO standards.^26^

In the 1993 Pelotas cohort, the 55-item Early Childhood Home Observation for Measurement of the Environment (EC-HOME) inventory was done in a subsample (n=632) at age 4 years.^27^ Items were scored as 0 or 1 if absent or present, respectively, as observed by trained data collectors or reported by mothers. Sample items were learning subscale^27^ (ie, “Child is helped to learn shapes and sizes at home”) and language subscale (ie, “The caregiver sings to the child daily”). We summed the two subscales in 18-item learning opportunities scores. The items evaluating responsiveness included verbal caregiver–child interactions (eg, “Parent converses with child at least twice during visit”). We summed the items to create 7-item responsive caregiving scores, as predetermined by the EC-HOME. In the Bt20+ cohort, responsive caregiving and learning opportunities were assessed at age 2 years (n=1838) with age-appropriate questions similar to EC-HOME. Responsiveness was based on a 6-item questionnaire from the EC-HOME completed by interviewer observation (eg, “Does the child appear happy, confident, and secure in the mother's presence?”). Learning opportunities were based on a 5-item questionnaire (eg, “Is there anything you are trying to teach your child at the moment?”). All measures were coded with higher scores denoting higher nurturance.

Early adversities were mother–child assessments during the perinatal period, infancy, and early childhood in the 1993 Pelotas and Bt20+ cohorts, before assessments of the nurturing care components.^2^ Measures were chosen a priori based on developmental theory^6^ and availability in the two datasets. No available adversity measures were excluded. Each measure contributed equally to the index. The environmental cumulative adversities index included: low-income household (lowest two wealth quintiles within each site); low maternal schooling (grades of schooling attainment below 60th percentile); short maternal stature (<150·1 cm, representing −2 height-for-age Z score below international standards^28^); maternal age at child's birth (<18 years); poor maternal mental health (self-report questionnaire in the 1993 Pelotas cohort [>7 points] at child age 4 years,^29^ and 24-item Pitt Depression Inventory in the Bt20+ cohort [≥20 points^30^] at child age 6 months); and household crowding (more than three people per room, UN threshold). Each child's environmental cumulative adversities index was summed, ranging from 0–6 points. The child cumulative adversities index included: low birthweight (<2500 g), preterm birth (<37 weeks), and stunted growth at 12 months (length-for-age Z score less than −2 relative to international standards^26^). The child cumulative adversities index ranged from 0–3 points. The total early cumulative adversities index is the sum of the environmental and child adversities (range 0–9), with higher scores denoting more adversities.

### Analytical sample

We restricted analysis to participants with responsive caregiving and learning opportunities data (1993 Pelotas cohort n=632; Bt20+ cohort n=1838; in appendix 2 p 1). In the 1993 Pelotas cohort, the EC-HOME inventory was done in a random subsample^14^ that included all low birthweight children (n=510) plus 20% of remaining children. All analyses accounted for the sampling weights. In the Bt20+ cohort, missingness was due to high urban mobility, with no statistically significant differences in birth outcomes between children retained through adolescence versus lost to follow-up.^31^ Our final analytical datasets for IQ were 547 and 1081; for psychosocial adjustments were 632 and 767; and for height-for-age Z score were 539 and 1130 in the 1993 Pelotas and Bt20+ cohorts, respectively.

### Data analysis

Z scores were created to allow comparison across sites on variables with different metrics in regression models, including psychosocial adjustments, responsive caregiving, learning opportunities, and environmental, child, and total cumulative adversities scores. Constructs were comparable across datasets, but were not pooled due to measurement and contextual differences. Variables were standardised to not force linearity in models testing interaction between adversities scores and nurturing variables. in Appendix 2 (p 2) provides the standardised values in mean Z scores of the analytical sample in addition to the descriptive statistics for the 1993 Pelotas and Bt20+ cohort. Full information maximum likelihood estimation was used to account for missing information in the cumulative adversities score items (maternal height, mental health, and child length-for-age Z score at 12 months were missing for 415 [30%], 526 [38%], and 615 [44%] participants, respectively, in the Bt20+ cohort). Other cumulative adversities items had less than 2% missing data. Models using listwise deletion yielded similar results, indicating that data were missing completely at random (in appendix 2 pp 8–10). Continuous variables were tested for differences between included and excluded cases using linear regression models. In the 1993 Pelotas and Bt20+ cohorts, participants with valid pre-school data were very similar to those with missing data (in appendix 2 p 3). We observed differences between included and excluded cases in maternal age in both sites; therefore, we did a sensitivity analysis to account for such differences in maternal age.

We used multivariable linear regression models with full information maximum likelihood to examine associations between early cumulative adversities and adolescent human capital controlling for child sex. Multivariable linear regression models were used to examine associations between responsive caregiving and learning opportunities and adolescent human capital, controlling for total cumulative adversities. To examine whether responsive caregiving and learning opportunities modified associations between cumulative adversities and adolescent human capital, we included interaction terms between the cumulative adversities scores (total, environmental, and child) and the nurturing home environment score. If the interaction term was statistically significant, we plotted the moderating variable (nurturing) as low, medium, or high (ie, −2 Z score, mean, +2 Z score, respectively), and tested the slope of the predictor variable (adversities) to identify the association driving the interaction. Our model specification checks, including assessment of model residuals, revealed that all normality assumptions were met with continuous outcomes. Effect modification by sex was tested in all models with a three-way interaction term among nurturing variables, cumulative adversities scores, and sex. These interaction terms were not statistically significant; thus pooled results are presented, adjusted for sex. Statistical analyses were done using Stata, version 15.1.

### Role of the funding source

The funders of the study had no role in study design, data collection, data analysis, data interpretation, or report writing. All authors had full access to all the data in the study and had final responsibility for the decision to submit for publication.

## Results

Mean environmental cumulative adversities scores were 1**·**4 (SD 1**·**1) in the 1993 Pelotas cohort and 1**·**7 (1**·**2) in the Bt20+ cohort (table 1). The number of children born into crowded households was higher in the Bt20+ cohort (618 [45%] of 1364) than in the 1993 Pelotas cohort (29 [4%] of 623). Mothers' schooling averaged 6**·**7 grades in the 1993 Pelotas cohort and 9**·**7 grades in the Bt20+ cohort. Mean total cumulative adversities were 1·6 points (SD 1·3) in the 1993 Pelotas cohort and 1·9 (1·4) in the Bt20+ cohort.

**Table 1:** Sample characteristics of the Pelotas and Bt20+ cohorts in Soweto, Johannesburg

		Pelotas*(n=547)	**Bt20+ (n=1081)**
**Human capital in adolescence**			
IQ standardised†		99·9 (14·7), 547	99·8 (14·9), 1081
Better psychosocial adjustments, sum score		4·9 (4·0), 632	20·6 (10·7), 767
WHO height-for-age Z score		–0·27 (1·0), 539	–0·66 (0·9), 1130
**Components of the nurturing care**‡			
Learning opportunities, sum score		9·4 (3·2), 632	7·9 (2·0), 1369
Responsive caregiving, sum score		5·7 (1·4), 632	8·6 (2·5), 1369
**Early adversities**			
Cumulative adversities: environmental§		1·4 (1·1), 598	1·7 (1·2), 716
	Lowest two wealth quintiles at birth	41·4%, 265	33·7%, 470
	Maternal schooling in grades attained¶	6·7 (3·6), 350	9·7 (2·6), 811
	Low maternal height (<150·1 cm)‖	8·4%, 60	8·8%, 85
	Maternal age at birth (≤18 years)	6·4%, 44	8·0%, 111
	Maternal mental health (poor)	25·5%, 173	24·8%, 212
	Crowded household (>three people per room)	4·4%, 29	45·3%, 618
Cumulative adversities: child**		0·2 (0·5), 627	0·3 (0·7), 758
	Low birthweight (<2500 g)	9·9%, 188	11·1%, 154
	Prematurity: preterm (<37 weeks)	6·3%, 69	12·7%, 177
	Stunted growth at 12 months (length-for-age Z score <2 SD)	8·8%, 84	10·6%, 81
Cumulative adversities: total††		1·6 (1·3), 596	1·9 (1·4), 429

Data are mean (SD), n or %, n. Pelotas=1993 Pelotas Birth Cohort. Bt20+=Birth to Twenty Plus Birth Cohort. IQ=intelligence quotient.

* Prevalence for Pelotas is weighted to correct for the oversampling of low birthweight children in preschool-age measurement.

† Mean 100 (SD 15).

‡ Sum score of the quality of the nurturing home environment.

§ Cumulative adversities: environmental is a sum of maternal and household characteristics (wealth, maternal schooling, maternal height, maternal age, maternal mental health, and household crowding), range 0–6.

¶ Mean (SD) of years of schooling self-reported by mothers, and total sample (n) below the 60th percentile.

‖ Reference to –2 height-for-age Z score relative to international standards.

** Cumulative adversities: child is a sum of a child's characteristics (birthweight, gestational age, and growth at 12 months), range 0–3.

†† Cumulative adversities: total is a sum of all early cumulative adversities, range 0–9.

Higher cumulative adversities scores predicted lower adolescent human capital in both the 1993 Pelotas and Bt20+ cohorts (table 2; in appendix 2 pp 4–7). For each additional point in total cumulative adversities score there was a decrease of 5·89 (95% CI −7·29 to −4·50) IQ points in the 1993 Pelotas cohort (p<0·0001) and a decrease of 2·69 (–4·52 to −0·86) IQ points in the Bt20+ cohort (p=0·0039). Similar associations were found between total cumulative adversities and psychosocial adjustments in the 1993 Pelotas cohort (–0·17 Z score [95% CI −0·30 to −0·05; p=0·007] for each additional point in total cumulative adversity score), but not significantly in the Bt20+ cohort (–0·07 [–0·24 to 0·09; p=0·379] Z score per point; in appendix 2 pp 4–5). Each additional point in total cumulative adversities was also associated with a decrease of 0·32 (95% CI −0·41 to −0·22; p<0·0001) adolescent height-for-age Z score in the 1993 Pelotas cohort and a decrease of 0·15 (–0·24 to −0·05; p=0·003) adolescent height-for-age Z score in the Bt20+ cohort; in appendix 2 pp 6–7).

**Table 2:** Association of early life cumulative adversities and home environment with adolescent IQ in the 1993 Pelotas and Birth to 20 Plus Birth Cohorts

		**Learning opportunities**	**Responsive caregiving**	**Child adversities**	**Environmental adversities**	**Total adversities**
IQ Pelotas (n=547)*						
Nurturing environment						
	Learning opportunities	6·73 (5·54 to 7·91)	··	··	··	··
	Responsive caregiving	··	3·35 (1·91 to 4·79)	··	··	··
Cumulative adversities						
	Environmental	··	··	··	–5·54 (–6·75 to –4·33)	··
	Child	··	··	–2·13 (–3·79 to –0·47)	··	··
	Total	··	··	··	··	–5·89 (–7·29 to –4·50)
Nurturing adjusted for adversities						
Learning opportunities						
	Environmental	5·30 (6·61 to 3·99)	··	··	–2·94 (–4·23 to –1·65)	··
	Child	6·64 (5·46 to 7·83)	··	–1·38 (–2·96 to 0·19)	··	··
	Total	5·47 (4·20 to 6·74)	··	··	··	–2·04 (–2·97 to –1·12)
Responsive caregiving						
	Environmental	··	2·01 (3·36 to 0·66)	··	–5·04 (–6·28 to –3·80)	··
	Child	··	3·34 (1·90 to 4·77)	–2·09 (–3·76 to –0·41)	··	··
	Total	··	2·26 (0·93 to 3·59)	··	··	–3·46 (–4·35 to –2·57)
IQ Bt20+ (n=1081)*						
Nurturing environment						
	Learning opportunities	1·20 (0·30 to 2·11)	··	··	··	··
	Responsive caregiving	··	0·64 (–0·30 to 1·58)	··	··	··
Cumulative adversities						
	Environmental	··	··	··	–2·68 (–4·10 to –1·26)	··
	Child	··	··	–2·69 (–4·52 to –0·86)	··	··
	Total	··	··	··	··	–2·69 (–4·52 to –0·86)
Nurturing adjusted for adversities						
Learning opportunities						
	Environmental	0·93 (0·01 to 1·86)	··	··	–2·62 (–4·04 to –1·20)	··
	Child	1·14 (2·05 to 0·22)	··	–0·75 (–1·93 to 0·42)	··	··
	Total	0·86 (–0·12 to 1·83)	··	··	··	–2·69 (–0·86 to –4·52)
Responsive caregiving						
	Environmental	··	0·65 (–0·30 to 1·59)	··	–2·67 (–4·09 to –1·25)	··
	Child	··	0·66 (1·60 to –0·27)	–0·81 (–1·99 to 0·36)	··	··
	Total	··	0·65 (–0·32 to 1·61)	··	··	–2·67 (–0·84 to –4·50)

Data are linear regression unstandardised coefficient (95% CI). IQ=intelligence quotient. Cumulative adversities: child is a sum of a child's characteristics (birthweight, gestational age, and growth at 12 months), range 0–3. Cumulative adversities: environmental is a sum of maternal and household characteristics (wealth, maternal schooling, maternal height, maternal age, maternal mental health, and household crowding), range 0–6. Cumulative adversities: total is a sum of all early adversities, range 0–9. Cumulative adversities (environmental, child, total) were analysed separately.

* Each row is a model. The sample size is the same across all models.

Childhood responsive caregiving and learning opportunities were positively associated with all three outcomes in both cohorts (table 2; in appendix 2 pp 4–7). In the 1993 Pelotas cohort, each additional Z score of learning opportunities was associated with 5·47 (95% CI 4·20 to 6·74) IQ points after adjusting for total cumulative adversities (table 2), and each additional Z score of responsive caregiving was associated with 2·26 (0·93 to 3·59) IQ points, independent from total cumulative adversities (table 2). However, these associations were not significant in the Bt20+ cohort (0·86 [–0·12 to 1·83] IQ points per Z score of learning opportunities and 0·65 [–0·32 to 1·61] IQ points per Z score of responsive caregiving; table 2). In the 1993 Pelotas cohort, each additional Z score of responsive caregiving was associated with −0·12 Z score of psychosocial adversities (–0·12 [–0·23 to −0·01]) after adjusting for cumulative adversities (in appendix pp 4–5). Learning opportunities were associated with greater adolescent height in the Bt20+ cohort (0·06 [95% CI 0·01 to 0·12; p=0·037] height-for-age Z score per Z score of learning opportunities), after accounting for total cumulative adversities, but not significantly in the 1993 Pelotas cohort (0·01 [–0·10 to 0·12]; in appendix 2 pp 6–7).

**Figure 2:**
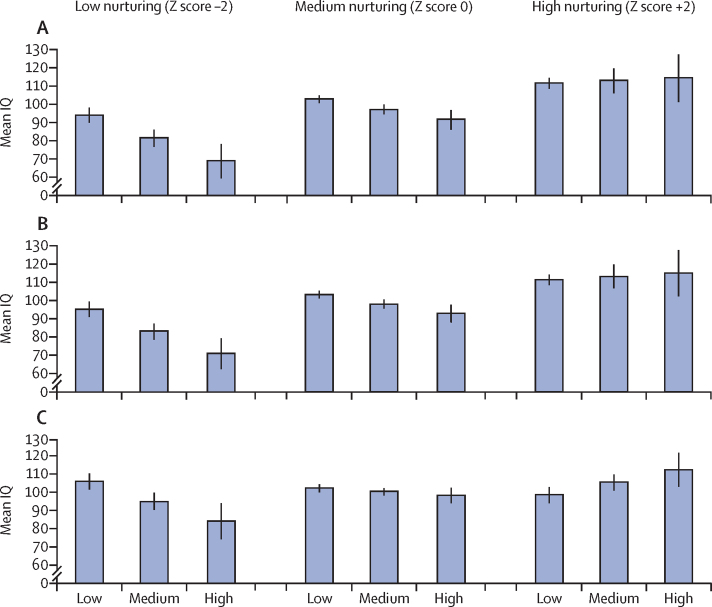
Model-adjusted mean IQ by nurturing environment and cumulative adversities Means are adjusted for child age, nurturing environment, cumulative adversities, and nurturing environment x cumulative adversities interaction. High, medium, and low adversity represent values at −1 Z score, +1 Z score, and +3 Z score of the adversity variable, respectively. (A) Learning opportunities and total adversities (Pelotas). (B) Learning opportunities and environmental adversities (Pelotas). (C) Responsive care giving and child adversities (Bt20+). Error bars represent 95% CIs. IQ=intelligence quotient. Pelotas=1993 Pelotas Birth Cohort. Bt20+=Birth to Twenty Plus Birth Cohort.

In the 1993 Pelotas cohort, the association between total cumulative adversities and IQ varied by learning opportunities (beta coefficient for interaction 1·74, 95% CI 0·43–3·04; figure 2; table 3). Under conditions of low-nurturing and medium-nurturing environment in terms of learning opportunities, there was a significant inverse association between total cumulative adversities and adolescent IQ (low beta coefficient −6**·**66, SE 1**·**88, p<0**·**0001; medium beta coefficient −3**·**18, 1**·**46, p=0**·**030); ie, each additional Z score of total cumulative adversity is associated with −6·66 IQ points in the low-nurturing environment. Under conditions of high nurturing in terms of learning opportunities, the association was not statistically significant (p=0·89; table 3). There was a similar pattern with environmental adversities. In the Bt20+ cohort, responsive caregiving modified the association of early child cumulative adversities with IQ (beta coefficient for interaction 2·24, 95% CI 0·94–3·54; figure 2); under conditions of low nurturing in terms of responsive caregiving, the association between child adversities and adolescent IQ was inverse and statistically significant (beta coefficient −5·4, SE 2·3; p=0**·**020), but was non-significant under conditions of medium and high responsive caregiving (figure 2; table 3).

**Table 3:** Interactions between adolescent IQ and adversities, and modifying effects of nurturing care in the home

	**Interaction**	**Nurturing environment**		
		Low	Medium	High
Learning and total adversities (Pelotas)	1·74 (0·66), 95% CI 0·43–3·04, p=0·0092	–6·66 (1·88), p<0·0001	–3·18 (1·46), p=0·030	0·30 (2·06), p=0·89
Learning and environmental adversities (Pelotas)	1·73 (0·63), 0·47–2·98, p=0·0070	–6·46 (1·75), p<0·0001	–3·01 (1·39), p=0·032	0·45 (2·0), p=0·82
Responsive caregiving and child adversities (Bt20+)	2·24 (0·66), 95% CI 0·94–3·54, p=0·0075	–5·4 (2·3), p=0·020	–0·95 (1·79), p=0·59	3·53 (2·14), p=0·099

Data are b (SE), 95% CI, p value, or b (SE) p value. Low, medium, or high nurturing environments represent values at –2 Z score, mean, and +2 Z score, respectively. Pelotas=1993 Pelotas Birth Cohort. Bt20+=Birth to Twenty Plus Birth Cohort. IQ=intelligence quotient.

There were no significant interactions between responsive caregiving or learning opportunities, and early cumulative adversities on psychosocial adjustments or height in the 1993 Pelotas or Bt20+ cohorts (in appendix pp 9–10). Our sensitivity analysis accounting for differences in maternal age (in appendix 2 pp 11–13) yielded similar results as our final models.

## Discussion

Three major findings emerged from this investigation of associations among early cumulative adversities, aspects of nurturing care, and adolescent human capital in two longitudinal studies in middle-income countries. First, the accumulation of early adversities was inversely associated with three domains of adolescent human capital: IQ, psychosocial adjustments, and height in the 1993 Pelotas cohort and with IQ and height in the Bt20+ cohort. These findings are consistent with evidence that cumulative adversities have long-term effects on physical and mental health and wellbeing.^32^ As other studies have shown, these associations might be explained by disruptions to adaptive functioning associated with accumulation of adversities.^33^ One possible explanation for sample differences in associations is that the Bt20+ cohort has an over-representation of low-income families, possibly limiting variability to detect differences, whereas the 1993 Pelotas cohort enrolled a diverse socioeconomic population. Another possibility is that while similar constructs were assessed across cohorts, the precise measures differed, and some were administered at differing ages.

Second, in both cohorts, responsive caregiving and learning opportunities during pre-school ages were positively associated with adolescent human capital, even after adjusting for early adversities, consistent with predictions from nurturing care.^2^, ^8^ The positive associations over more than a decade, across two sets of youth, and based on multiple measurement methods show the strength of the conceptual model linking nurturing care to adolescent human capital. The positive association of learning opportunities with adolescent height in the Bt20+ cohort might indicate the presence of other positive nurturing care components (eg, health, nutrition, and safety and security) in the home. A review of longitudinal studies concluded that height is more strongly associated with early health and nutrition than with early responsive caregiving and learning opportunities.^7^

Third, the association between cumulative adversities and adolescent human capital differed depending on nurturing (responsive caregiving and learning opportunities). In both settings, a nurturing early home environment during pre-school ages mitigated negative effects of early cumulative adversities on adolescent IQ. The absence of differences by cumulative adversities in adolescent IQ scores in a high nurturing environment suggests a protective mechanism that enabled children to learn and develop in spite of early adversities. A nurturing home environment has also been shown to protect children's early development from negative associations with stunting.^10^ Our results extend findings from earlier studies^9^ by suggesting that the beneficial associations for parenting programmes found among children with stunting extend to cumulative adversities. Our findings corroborate previous studies from high-income countries suggesting that responsive caregiving protects against other poverty-related threats to child health, development, and wellbeing.^34^ Evidence from both high-income countries and LMICs show that nurturing home environments buffer children exposed to early cumulative adversities against negative human capital outcomes, thereby lessening income inequalities.^35^

Adolescents had the lowest IQ scores in the context of high cumulative adversities and low nurturing home environment. The observational evidence that a nurturing home environment attenuated the negative effects of cumulative adversities suggests that responsive caregiving and learning opportunities during the pre-school years advanced the developmental course toward higher adolescent IQ, particularly among children with early cumulative adversities. If, in future intervention trials, the impact of nurturing care interventions is shown to vary by cumulative adversities, this finding would provide additional evidence to support the implementation of nurturing care policies and programmes to mitigate the negative effects of early adversities throughout the life course.

The mitigating effects of responsive caregiving and learning opportunities on associations between early cumulative adversities and adolescent human capital differed between the two cohorts. In the 1993 Pelotas cohort, the associations were mitigated by learning opportunities and in the Bt20+ cohort by responsive caregiving. Both responsive caregiving and learning opportunities have been associated with cognitive development in multiple studies.^7^ Their association with cognition might vary by timing and context. In the Bt20+ cohort, nurturing environment was measured at age 2 years and in the 1993 Pelotas cohort at age 4 years. Differences in pre-schoolers' independence, motor skills, and cognitive and language development might have elicited different levels of responsive caregiving and learning opportunities.

We did not find evidence that either responsive caregiving or learning opportunities buffered the negative effects of early cumulative adversities on psychosocial adjustment or height. Our findings are in line with a meta-analysis of early interventions showing positive associations of responsive care and learning opportunities with children's cognition, but not with height.^7^ Integrated nutrition and learning interventions have shown promising effects on growth and cognitive development among children experiencing early cumulative adversities.^36^ Although limited catch-up growth might occur after the first 1000 days, interventions that use a life course approach starting in the first 1000 days, and support enabling environments that promote all components of nurturing care, are recommended to promote healthy growth and development.^37^

A major strength of this study was the use of prospective data on early adversities, home nurturing care, and adolescent human capital measured at similar ages in large, longitudinal, population-based studies in South Africa and Brazil. We determined the specificity of two components of nurturing care during the pre-school years that mitigated early adversities on IQ (not on psychosocial adjustment and height) using three proxies of human capital development. By examining data from two diverse settings, we showed consistency of the associations, thus increasing the external validity of our findings.

The following limitations should be acknowledged. Cumulative adversity models do not typically consider the duration or intensity of adversities throughout childhood. Our model did not account for frequent or prolonged experiences of early adversities (ie, maternal mental health, maternal education, or wealth) or caregiver–child feedback loops that could undermine caregivers' provision of responsive care and early learning. To the extent that the families' environment predicts both child adversity and nurturing care, our models might have underestimated the potential for external interventions to promote components of nurturing care to mitigate the consequences of early adversities. This Article presents within-sample analysis because the two birth cohorts were not designed to be companions, and instruments and timing of measures differ. Because of unobserved factors and unmeasured confounding, including important aspects of the environment, such as maternal and paternal IQ that were not collected, our results cannot infer causality. Lastly, the analysis might have been underpowered in the interaction models, although our estimated coefficients of the interactions were robust using a p value cutoff of less than 0·05. Adjustments for multiple testing were not made, because our analyses were done on a priori hypotheses, and our dependent variables (IQ, psychosocial adjustments, and height-for-age Z score) were unique and not confounded by method invariance. Despite these caveats, the study shows that responsive caregiving and learning opportunities in the home can mitigate associations between cumulative adversities and adolescent cognitive development in two middle-income countries.

In summary, we found that two components of nurturing care, namely responsive caregiving and learning opportunities, were associated with increased adolescent human capital in the domains of cognition, psychosocial adjustment, and height, and might protect adolescent IQ from negative trajectories associated with early cumulative adversities. Furthermore, opportunities for responsive caregiving and early learning might be particularly beneficial for children with the greatest cumulative adversities. Parenting interventions have effectively modified both responsive caregiving and early learning in both LMICs^35^ and high-income countries,^34^ suggesting that gaps in human capital development can be reduced globally by programmes that enhance home nurturing care.^8^ This encouraging finding can inform future programmes and services necessary to achieve the Sustainable Developmental Goals and help ensure that no child is left behind.^38^

## Data sharing

Please contact Ana Menezes (anamene.epi@gmail.com) and Linda Richter (Linda.Richter@wits.ac.za) regarding data requests.

## Supplementary Materials

Portuguese translation of the abstract
