# Supplementary material for: Effects of responsive caregiving and learning opportunities during pre-school ages on the association of early adversities and adolescent human capital: an analysis of birth cohorts in two middle-income countries
Source: Lancet Child Adolesc Health. 2021 Jan;5(1):37–46. doi: 10.1016/S2352-4642(20)30309-6 (PMC7763480; doi:10.1016/S2352-4642(20)30309-6)
Supplement: Portuguese translation of the abstract [file mmc1.pdf]

THE LANCET
Child & Adolescent Health

Supplementary appendix 1

This translation in Portuguese was submitted by the authors and we reproduce it as supplied. It has not been peer reviewed. The Lancet's editorial processes have only been applied to the original in English, which should serve as reference for this manuscript.

Esta tradução em português foi submetida pelos autores e nós não fizemos quaisquer alterações. Esta versão não foi revista por pares. O processo editorial do The Lancet só foi aplicado à versão original em inglês, que deve servir como referência para este artigo.

Supplement to: Trude ACB, Richter LM, Behrman JR, et al. Effects of responsive caregiving and learning opportunities during pre-school ages on the association of early adversities and adolescent human capital: an analysis of birth cohorts in two middle-income countries. *Lancet Child Adolesc Health* 2021; **5:** 37–46.

Resumo

Introdução: Milhões de crianças no mundo estão sob risco de não desenvolverem todo seu potencial devido às adversidades no início da vida. Os cuidados responsivos e as oportunidades de aprendizagem, componentes do Plano de Cuidados de Criação (*Nurturing Care Framework*), podem mitigar os efeitos das adversidades.

Métodos: Analisamos dados longitudinais de coortes de nascimentos do Brasil (Pelotas 1993, n=632) e da África do Sul (Bt20+, n=1130) para avaliar se os cuidados responsivos e as oportunidades de aprendizagem modificam as associações entre o índice cumulativo de adversidades no início da vida e o capital humano do adolescente. O índice cumulativo de adversidades incluiu nove fatores: renda familiar; aglomeração na residência; escolaridade, altura, idade e saúde mental materna; peso ao nascer, idade gestacional e comprimento aos 12 meses da criança. Extraímos os dados sobre cuidados responsivos/oportunidades de aprendizagem do Inventário HOME (Pelotas: 4 anos; Bt20+: 2 anos). Examinamos três indicadores de capital humano: cognição, QI (Pelotas: 18 anos; Bt20+: 16 anos); ajuste psicossocial (Pelotas: 15 anos; Bt20+: 14 anos); e altura (Pelotas: 18 anos; Bt20+: 16 anos). Utilizamos modelos lineares com termos de interação entre índice cumulativo de adversidades e cuidados responsivos/oportunidades de aprendizagem para predizer o capital humano do adolescente.

Resultados: Para cada ponto adicional no escore-z de adversidades cumulativas, o QI na adolescência diminuiu 5,9 pontos em Pelotas ($p<0,0001$) e 2,7 pontos em Bt20+ ($p=0,0039$), ajustes psicossociais e escores-z de altura para idade diminuíram. Oportunidades de aprendizagem foram associadas com um aumento de 6,7 pontos no QI por escore-z em Pelotas ($p<0,0001$) e 1,2 em Bt20+ ($p=0,0089$). Associações entre adversidades cumulativas e QI foram modificadas por oportunidades de aprendizagem em Pelotas (b-interação: 1,74 - 95% CI 0,45; 3,08; $p=0,0092$) e por cuidados responsivos em Bt20+ (b-interação: 2,24 - 95% CI 0,92; 3,37; $p=0,0075$). Um ambiente domiciliar altamente estimulante atenuou os efeitos negativos de adversidades cumulativas no QI. Oportunidades de aprendizagem e cuidados responsivos não modificaram as associações de adversidades com ajustes psicossociais e altura do adolescente.

Interpretação: Ambientes domiciliares estimulantes no início da vida protegem as crianças contra os efeitos das adversidades no início da vida no QI na adolescência, com associações positivas de longo prazo na cognição do adolescente em dois países de renda média.

Financiamento: Fundação Bill e Melinda Gates OPP1148933

Palavras-chave: Plano de Cuidados de Criação; Capital Humano do Adolescente; Adversidades Cumulativas; Mitigação; Coortes de Nascimentos; Países de Renda Média
