# Supplementary material for: Effects of responsive caregiving and learning opportunities during pre-school ages on the association of early adversities and adolescent human capital: an analysis of birth cohorts in two middle-income countries
Source: Lancet Child Adolesc Health. 2021 Jan;5(1):37–46. doi: 10.1016/S2352-4642(20)30309-6 (PMC7763480; doi:10.1016/S2352-4642(20)30309-6)
Supplement: Supplementary appendix 2 [file mmc2.pdf]

THE LANCET
Child & Adolescent Health

Supplementary appendix 2

This appendix formed part of the original submission and has been peer reviewed. We post it as supplied by the authors.

Supplement to: Trude ACB, Richter LM, Behrman JR, et al. Effects of responsive caregiving and learning opportunities during pre-school ages on the association of early adversities and adolescent human capital: an analysis of birth cohorts in two middle-income countries. *Lancet Child Adolesc Health* 2021; **5:** 37–46.

Supplementary Materials

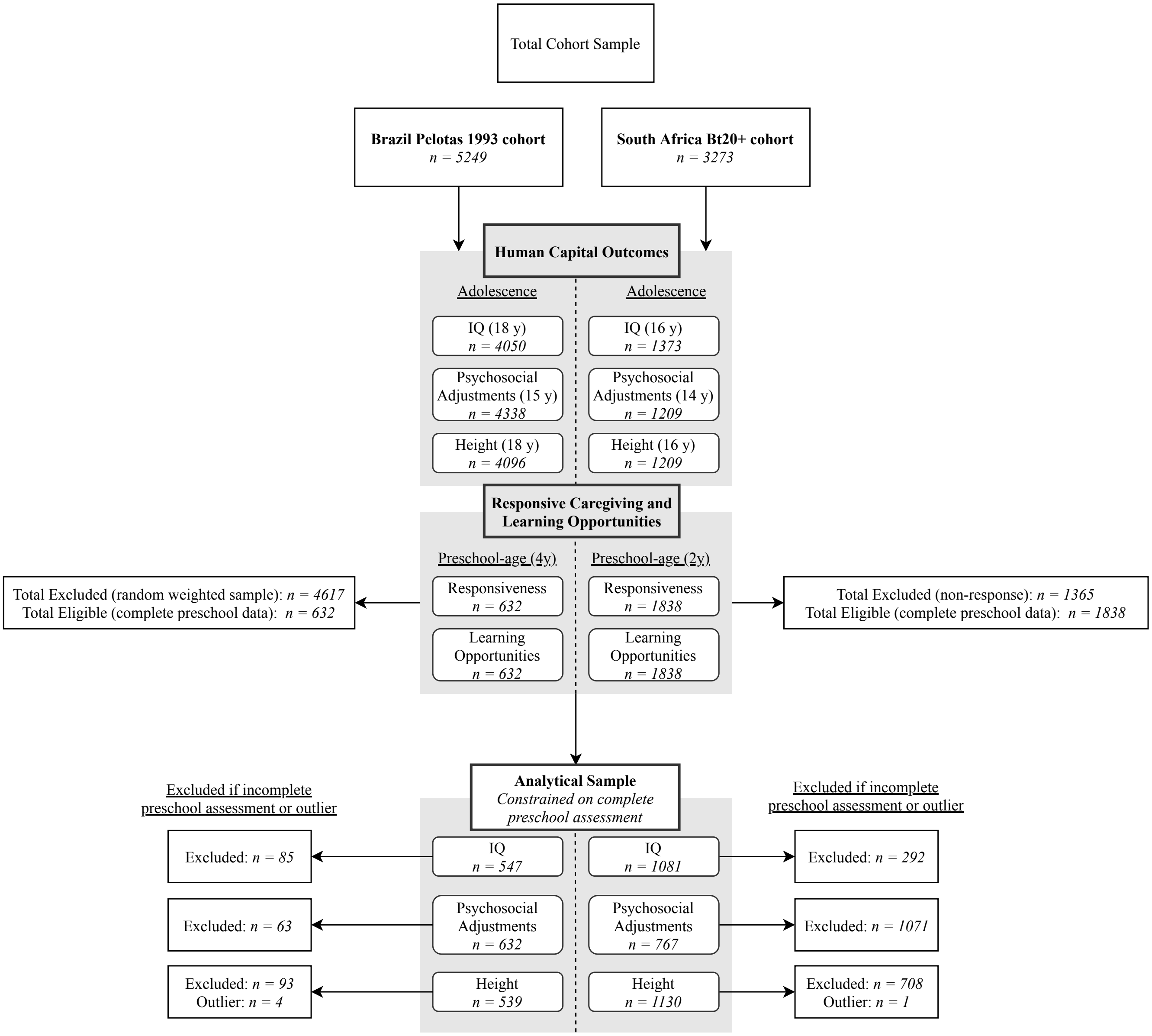

Supplementary Figure S1. Detailed participant flow diagram of the analytical sample of the 1990 Bt20+ and the 1993 Pelotas cohort studies.

Supplementary Table S1. Average Z-score of main variables in the analytical sample and demographic characteristics of all cases in both sites.

	Pelotas*		Soweto	
Analytical Sample (Z-score variables)	Mean (SD)	n	Mean (SD)	n
Learning Opportunities, Z-score	0·04 (1·02)	632	-0·05 (0·97)	1392
Responsiveness, Z-score	-0·00 (1·00)	632	0·03 (0·96)	1392
IQ 18 years old standardized (mean 100 and SD 15)[1]	0·02 (0·98)	547	-0·00 (1·00)	1081
Better Psychosocial Adjustments, Z- score[2]	-0·02 (0·93)	632	0·02 (1·02)	767
WHO height-for-age Z-score[3]	-0·27 (1·01)	539	-0·66 (0·91)	1130
Cumulative Adversities: Environmental, Z-score	-0·07 (0·98)	598	0·01 (0·99)	594
Cumulative Adversities: Child, Z-score	-0·36 (0·74)	627	0·04 (1·01)	758
Cumulative Adversities: Total, Z-score	-0·23 (0·88)	596	-0·01 (1·01)	370
All available data				
Learning Opportunities Score, Z-score	0·04 (1·02)	632	-0·00 (1·00)	1838
Responsive Caregiving Score, Z-score	-0·00 (1·00)	632	-0·00 (1·00)	1838
IQ 18 years old standardized (mean 100 and SD 15)[1]	0·02 (1·00)	4050	0·00 (1·00)	1612
Avoidance of problem behaviors, Z- score[2]	0·02 (1·00)	5249	-0·00 (1·00)	1209
WHO height-for-age Z-score[3]	-0·11 (0·96)	4095	-0·63 (0·92)	1610
Cumulative Adversities: Environmental, Z-score	-0·07 (0·98)	598	0·00 (1·00)	763
Cumulative Adversities: Child, Z-score	-0·36 (0·72)	1352	-0·00 (1·00)	1386
Cumulative Adversities: Total, Z-score	-0·23 (0·88)	596	-0·00 (1·00)	451
Early Adversities (%)				
Lowest two wealth quintile at birth[5]	42·9	2226	32·5	603
Maternal years of schooling (bellow 60th percentile)[6]	52·7	2815	58·7	1095
Low maternal height (<150·1 cm)[7]	8·0	444	8·70	123
Maternal age at birth (≤ 18 years)	8·1	429	7·4	150
Maternal mental health (poor)[8]	25·5	173	25·2	293
Crowded household (> 3 persons/room)[9]	4·9	59	43·9	763
Low birthweight (< 2500g)	2·7	510	11·0	223
Prematurity: preterm (<37 weeks)	5·6	381	12·9	257
Stunted growth at 12 months (HAZ <2 SD)	9·0	176	4·8	98

Abbreviations: SD (standard deviation); IQ (intelligence quotient); WHO (World Health Organization); HAZ (height-for-age Z-score)

* Prevalence for Pelotas is weighted to correct for the oversampling of low birthweight children in preschool-age measurement.

[1] IQ measured in Pelotas with the WAIS-III at 18 years and in Bt20+ with Ravens at 16 years; [2] Pelotas used the Strengths and Difficulty Questionnaire at 15 years and Bt20+ the Youth Self-Report at 14 years; [3] Height was assessed at 18 years in Pelotas and at 16 years in Bt20+; [4] Sum score of the quality of the nurturing home environment; [5] Lowest two quintile; [6] Mean and SD of years of schooling self-reported by mothers, and total sample (n) below the 60th percentile; [7] Referent to -2 HAZ relative to international standards; [8] Defined in Pelotas as >7 points in the Self Report Questionnaire (SRQ-20) and ≥ 20 points on the Pitt Inventory; [9] According to the United Nations threshold.

Supplementary Table S2. Differences in main variables between included and excluded cases with complete early childhood information.

	Included		Excluded		
Variable Name	n	Mean (SD)	n	Mean (SD)	p-value
**1993 Pelotas**					
Main variables					
IQ 18 years old standardized mean 100 and SD 15	547	99·9 (14·67)	3503	100·01 (15·03)	0·914
Better Psychosocial Adjustments, Sum score	631	4·98 (4·03)	4617	5·17 (4·35)	0·309
WHO Height-Z-score 15 years	539	-0·27 (1·01)	3556	-0·08 (0·95)	<0·001
Cumulative Adversities: Environmental, Sum score	598	1·66 (0·05)	0	··	··
Cumulative Adversities: Child, Sum score	627	0·25 (0·01)	725	0·25 (0·01)	0·876
Cumulative Adversities: Total, Sum score	596	1·90 (0·05)	0	··	··
Covariates					
Maternal age	632	26·92 (6·42)	4616	25·90 (6·36)	<0·001
Maternal years of schooling	630	6·95 (3·70)	4612	6·80 (3·61)	0·384
Maternal height	627	159·71 (7·10)	4576	159·97 (6·67)	0·424
Wealth quintile at birth	623	2·98 (1·39)	4514	2·97 (1·43)	0·885
Child birthweight (grams)	632	3202·82 (541·53)	4600	3242·64 (460·24)	0·069
Gestational age (weeks)	569	39·69 (2·33)	4103	39·67 (2·17)	0·850
Length-for-age z-score at 12 months	629	-0·28 (1·29)	732	-0·22 (1·28)	0·431
**Bt20+ Soweto**					
IQ 18 years old standardized mean 100 and SD 15	1080	99·98 (14·96)	1612	100·04 (15·00)	0·846
Better Psychosocial Adjustments, Sum score	767	20·61 (10·75)	1209	20·38 (10·56)	0·313
WHO Height-Z-score 15 years	1130	-0·65 (0·91)	1610	-0·63 (0·92)	0·115
Cumulative Adversities: Environmental, Sum score	594	1·73 (1·19)	716	1·72 (1·20)	0·985
Cumulative Adversities: Child, Sum score	758	0·35 (0·66)	967	0·35 (0·68)	0·421
Cumulative Adversities: Total, Sum score	370	1·96 (1·40)	429	1·97 (1·38)	0·445
Covariates					
Maternal age	1836	25·74 (6·13)	1435	26·26 (6·00)	0·001
Maternal years of schooling	1833	9·75 (2·84)	1099	9·23 (3·23)	0·561
Maternal height	1024	158·60 (6·51)	486	158·82 (6·40)	0·985
Wealth quintile at birth	1838	3·02 (1·32)	1022	2·88 (1·31)	0·897
Child birthweight (grams)	1834	3083·29 (509·93)	1433	3054·52 (516·27)	0·316
Gestational age (weeks)	1798	38·08 (1·90)	1373	38·23 (1·94)	0·123
Length-for-age z-score at 12 months	1141	-1·29 (1·25)	0	··	··

Abbreviations: SD (standard deviation); IQ (intelligence quotient); WHO (World Health Organization); HAZ (height-for-age Z-score)

·· All variables included

Supplementary Table S3. Association of early life cumulative adversities and home environment with psychosocial adjustments in the Pelotas 1993 and Birth to 20 Plus cohorts.

	Learning Opportunities	Responsive Caregiving	Child Adversities	Environmental Adversities	Total Adversities
**Psychosocial Adjustments Pelotas (n=632)[1]**	b (95% CI)	b (95% CI)	b (95% CI)	b (95% CI)	b (95% CI)
**Nurturing Environment**					
Learning Opportunities	0·10 (-0·01, 0·21)	··	··	··	··
Responsive Caregiving	··	-0·08 (-0·19, 0·03)	··	··	··
**Cumulative Adversities**					
Child	··	··	-0·19 (-0·31, -0·07)	··	··
Environmental	··	··	··	-0·02 (-0·09, 0·13)	··
Total	··	··	··	··	-0·17 (-0·30, -0·05)
**Nurturing Adjusted for Adversities**					
Learning Opportunities					
+ Child	0·10 (-0·01, 0·22)	··	0·04 (-0·08, 0·15)	··	··
+ Environmental	0·02 (0·15, -0·11)	··	··	-0·18 (-0·31, -0·04)	··
+ Total	0·05 (-0·08, 0·17)	··	··	··	-0·09 (-0·18, -0·00)
Responsive caregiving					
+ Child	··	-0·08 (-0·19, 0·03)	0·02 (-0·09, 0·13)	··	··
+ Environmental	··	-0·14 (-0·03, -0·25)	··	-0·23 (-0·35, -0·11)	··
+ Total	··	-0·12 (-0·23, -0·01)	··	··	-0·13 (-0·21, -0·05)
**Psychosocial Adjustments Bt20+ (n=767)[1]**					
**Nurturing Environment**					
Learning Opportunities	0·02 (-0·06, 0·10)	··	··	··	··
Responsive Caregiving	··	-0·04 (-0·12, 0·04)	··	··	··
**Cumulative Adversities**					
Child	··	··	0·02 (-0·10, 0·13)	··	··
Environmental	··	··	··	0·03 (-0·09, 0·16)	··
Total	··	··	··	··	-0·07 (-0·24, 0·09)

**Nurturing Adjusted for Adversities**					
Learning Opportunities					
+ Child	0·02 (0·11, -0·06)	··	0·02 (-0·09, 0·14)	··	··
+ Environmental	0·03 (-0·06, 0·11)	··	··	0·03 (-0·09, 0·16)	··
+ Total	0·01 (-0·08, 0·10)	··	··	··	-0·08 (0·09, -0·24)
Responsive caregiving					
+ Child	··	-0·04 (0·04, -0·12)	0·02 (-0·10, 0·13)	··	··
+ Environmental	··	-0·04 (-0·12, 0·04)	··	0·03 (-0·10, 0·15)	··
+ Total	··	-0·05 (-0·13, 0·04)	··	··	-0·08 (0·09, -0·25)

Abbreviations: IQ (intelligence quotient); b (linear regression unstandardized coefficient); CI (confidence interval)

[1]Each row is a model. The sample size is the same across all models.

Cumulative Adversities Child is a sum of child's characteristics (birthweight, gestational age, and growth at 12 months), range from 0-3.

Cumulative Adversities Environmental is a sum of maternal and household characteristics (wealth, maternal schooling, maternal height, maternal age, maternal mental health, and household crowding), range from 0-6.

Cumulative Adversities Total is a sum of all early adversities and range from 0-9.

Cumulative adversities (total, environmental, and child) were analyzed separately.

Supplementary Table S4. Association of early life cumulative adversities and home environment with adolescent height in the Pelotas 1993 and Birth to 20 Plus cohorts.

	**Learning Opportunities**	**Responsive Caregiving**	**Child Adversities**	**Environmental Adversities**	**Total Adversities**
**HAZ Pelotas (n=539)[1]**	b (95% CI)	b (95% CI)	b (95% CI)	b (95% CI)	b (95% CI)
**Nurturing Environment**					
Learning Opportunities	0·13 (0·03, 0·23)	··	··	··	··
Responsive Caregiving	··	0·12 (0·02, 0·22)	··	··	··
**Cumulative Adversities**					
Child	··	··	-0·48 (-0·57, -0·39)	··	··
Environmental	··	··	··	-0·16 (-0·26, -0·07)	··
Total	··	··	··		-0·32 (-0·41, -0·22)
**Nurturing Adjusted for Adversities**					
Learning Opportunities					
+ Child	0·09 (-0·00, 0·19)	··	-0·47 (-0·56, -0·38)	··	··
+ Environmental	0·07 (0·18, -0·04)	··	··	-0·13 (-0·23, -0·02)	··
+ Total	0·01 (-0·10, 0·12)	··	··	··	-0·20 (-0·27, -0·13)
Responsive caregiving					
+ Child	··	0·11 (0·01, 0·20)	-0·48 (-0·57, -0·39)	··	··
+ Environmental	··	0·08 (0·18, -0·02)	··	-0·14 (-0·24, -0·05)	··
+ Total	··	0·05 (-0·05, 0·15)	··	··	-0·20 (-0·26, -0·13)
**HAZ Bt20+ (n=1 130)[1]**					
**Nurturing Environment**					
Learning Opportunities	0·09 (0·03, 0·14)	··	··	··	··
Responsive Caregiving	··	0·02 (-0·04, 0·07)	··	··	··
**Cumulative Adversities**					
Child	··	··	-0·25 (-0·32, -0·19)	··	··
Environmental	··	··	··	-0·10 (-0·18, -0·01)	··
Total	··	··	··		-0·15 (-0·24, -0·05)
**Nurturing Adjusted for Adversities**					
Learning Opportunities					

+ Child	0·06 (0·11, 0·01)	··	-0·25 (-0·31, -0·18)	··	··
+ Environmental	0·07 (0·02, 0·13)	··	··	-0·09 (-0·18, -0·01)	··
+ Total	0·06 (0·00, 0·12)	··	··	··	-0·14 (-0·04, -0·24)
Responsive caregiving					
+ Child	··	0·02 (0·07, -0·04)	-0·25 (-0·32, -0·19)	··	··
+ Environmental	··	0·02 (-0·04, 0·07)	··	-0·10 (-0·18, -0·01)	··
+ Total	··	0·02 (-0·04, 0·07)	··	··	-0·15 (-0·05, -0·24)

Abbreviations: IQ (intelligence quotient); b (linear regression unstandardized coefficient); CI (confidence interval)

[1]Each row is a model. The sample size is the same across all models.

Cumulative Adversities Child is a sum of child's characteristics (birthweight, gestational age, and growth at 12 months), range from 0-3.

Cumulative Adversities Environmental is a sum of maternal and household characteristics (wealth, maternal schooling, maternal height, maternal age, maternal mental health, and household crowding), range from 0-6.

Cumulative Adversities Total is a sum of all early adversities and range from 0-9.

Cumulative adversities (total, environmental, and child) were analyzed separately

Supplementary Table S5: Sensitivity analysis of the IQ models using listwise deletion method.

	Cumulative Adversities		Nurturing		Adversities*Nurturing	
**IQ Pelotas (n=542)**	b1	p-value	b2	p-value	b3 (95% CI)	p-value
**Model 1: Base Analysis**						
Learning Opportunities	··	··	6·73 (5·54, 7·91)	<0·001	··	··
Responsive Caregiving	··	··	3·35 (1·91, 4·80)	<0·001	··	··
Cumulative Adversities: Total	-5·74 (-7·11, -4·37)	<0·001		··	··	··
Cumulative Adversities: Environmental	-5·41 (-6·61, -4·21)	<0·001		··	··	··
Cumulative Adversities: Child	-2·12 (-3·78, -0·47)	0·012		··	··	··
**Model 2: Nurturing Adjusted for Total Adversities**						
Learning Opportunities	··	··	5·17 (3·90, 6·45)	<0·001	··	··
Responsive Caregiving	··	··	2·08 (0·76, 3·41)	0·002	··	··
**Model 3: Learning Opportunities*Cumulative Adversities**						
Total	-2·81 (-4·26, -1·35)	<0·001	6·12 (4·64, 7·60)	<0·001	1·74 (0·44, 3·04)	0·009
Environmental	-2·60 (-3·92, -1·28)	<0·001	5·80 (4·31, 7·28)	<0·001	1·73 (0·47, 2·99)	0·007
Child	-1·35 (-2·91, 0·20)	0·088	6·66 (5·48, 7·83)	<0·001	0·20 (-1·06, 1·46)	0·758
**Model 4: Responsiveness*Cumulative Adversities**						
Total	-5·25 (-6·60, -3·90)	<0·001	2·05 (0·68, 3·41)	0·003	-0·22 (-1·67, 1·23)	0·765
Environmental	-4·94 (-6·17, -3·71)	<0·001	1·90 (0·56, 3·24)	0·005	-0·11 (-1·33, 1·11)	0·858
Child	-2·07 (-3·76, -0·38)	0·016	2·99 (1·32, 4·66)	<0·001	-0·60 (-2·74, 1·53)	0·581
**IQ Bt20+ (n=279 to 573)**						
**Model 1: Base Analysis**						
Learning Opportunities	··	··	1·20 (0·29, 2·11)	0·009	··	··
Responsive Caregiving	··	··	0·64 (-0·30, 1·58)	0·180	··	··
Cumulative Adversities: Total	-2·49 (-4·24, -0·74)	0·006	··	··	··	··
Cumulative Adversities: Environmental	-2·44 (-3·77, -1·11)	<0·001	··	··	··	··
Cumulative Adversities: Child	-0·85 (-2·08, 0·39)	0·179	··	··	··	··
**Model 2: Nurturing Adjusted for Total Adversities**						
Learning Opportunities	··	··	-0·15 (-1·98, 1·68)	0·869	··	··
Responsive Caregiving	··	··	-1·15 (-3·00, 0·70)	0·222	··	··
**Model 3: Learning Opportunities*Cumulative Adversities**						
Total	-2·43 (-4·19, -0·66)	0·007	-0·15 (-1·98, 1·68)	0·872	-1·16 (-3·04, 0·71)	0·223
Environmental	-2·39 (-3·73, -1·05)	<0·001	0·51 (-0·91, 1·93)	0·478	-0·30 (-1·74, 1·14)	0·679
Child	-0·74 (-1·99, 0·50)	0·242	0·66 (-0·65, 1·98)	0·322	0·52 (-0·65, 1·70)	0·382
**Model 4: Responsiveness*Cumulative Adversities**						
Total	-2·53 (-4·28, -0·78)	0·005	-0·67 (-2·66, 1·31)	0·504	1·25 (-0·63, 3·13)	0·192
Environmental	-2·55 (-3·89, -1·21)	<0·001	-0·56 (-2·03, 0·90)	0·450	1·00 (-0·46, 2·47)	0·179
Child	-0·98 (-2·21, 0·25)	0·117	0·33 (-0·95, 1·62)	0·609	2·24 (0·94, 3·54)	0·001

Supplementary Table S6: Sensitivity analysis of psychosocial adjustments models using listwise deletion method.

	Cumulative Adversities		Nurturing		Adversities*Nurturing	
**Psychosocial Adjustments Pelotas (n=627)**	b1	p-value	b2	p-value	b3 (95% CI)	p-value
**Model 1: Base Analysis**						
Learning Opportunities	··	··	0·10 (-0·01, 0·21)	0·079	··	··
Responsive Caregiving	··	··	-0·08 (-0·19, 0·03)	0·164	··	··
Cumulative Adversities: Total	-0·17 (-0·29, -0·05)	0·009	··	··	··	··
Cumulative Adversities: Environmental	-0·19 (-0·30, -0·07)	0·002	··	··	··	··
Cumulative Adversities: Child	0·02 (-0·09, 0·13)	0·580	··	··	··	··
**Model 2: Nurturing Adjusted for Total Adversities**						
Learning Opportunities	··	··	0·05 (-0·08, 0·18)	0·432	··	··
Responsive Caregiving	··	··	-0·12 (-0·24, -0·01)	0·029	··	··
**Model 3: Learning Opportunities*Cumulative Adversities**						
Total	-0·16 (-0·30, -0·01)	0·034	0·02 (-0·12, 0·16)	0·790	-0·06 (-0·20, 0·08)	0·375
Environmental	-0·19 (-0·33, -0·05)	0·008	-0·01 (-0·15, 0·13)	0·904	-0·08 (-0·22, 0·05)	0·221
Child	0·05 (-0·07, 0·16)	0·413	0·12 (0·01, 0·22)	0·032	0·04 (-0·06, 0·15)	0·387
**Model 4: Responsiveness*Cumulative Adversities**						
Total	-0·20 (-0·33, -0·08)	0·002	-0·12 (-0·23, -0·01)	0·036	0·04 (-0·10, 0·17)	0·592
Environmental	-0·23 (-0·35, -0·11)	<0.001	-0·14 (-0·26, -0·03)	0·013	0·00 (-0·12, 0·13)	0·963
Child	0·02 (-0·09, 0·13)	0·733	-0·05 (-0·16, 0·06)	0·368	0·08 (-0·04, 0·19)	0·203
**Psychosocial Adjustments Bt20+ (n=185 to 364)**						
**Model 1: Base Analysis**						
Learning Opportunities	··	··	0·02 (-0·06, 0·11)	0·632	··	··
Responsive Caregiving	··	··	-0·04 (-0·12, 0·04)	0·309	··	··
Cumulative Adversities: Total	-0·08 (-0·25, 0·10)	0·385	··	··	··	··
Cumulative Adversities: Environmental	0·03 (-0·10, 0·17)	0·616	··	··	··	··
Cumulative Adversities: Child	0·02 (-0·09, 0·13)	0·755	··	··	··	··
**Model 2: Nurturing Adjusted for Total Adversities**						
Learning Opportunities	··	··	-0·01 (-0·19, 0·18)	0·929	··	··
Responsive Caregiving	··	··	-0·13 (-0·32, 0·06)	0·166	··	··
**Model 3: Learning Opportunities*Cumulative Adversities**						
Total	-0·08 (-0·26, 0·09)	0·347	-0·03 (-0·21, 0·16)	0·782	0·13 (-0·05, 0·31)	0·144
Environmental	0·04 (-0·10, 0·17)	0·596	0·01 (-0·13, 0·16)	0·853	-0·02 (-0·17, 0·13)	0·820
Child	0·03 (-0·08, 0·14)	0·578	0·01 (-0·11, 0·14)	0·844	0·09 (-0·01, 0·19)	0·079
**Model 4: Responsiveness*Cumulative Adversities**						
Total	-0·08 (-0·25, 0·09)	0·351	-0·17 (-0·36, 0·02)	0·080	-0·17 (-0·36, 0·01)	0·068
Environmental	0·04 (-0·09, 0·18)	0·522	-0·10 (-0·24, 0·03)	0·134	-0·13 (-0·28, 0·01)	0·078
Child	0·02 (-0·09, 0·13)	0·744	-0·07 (-0·19, 0·04)	0·190	0·00 (-0·10, 0·10)	0·993

Supplementary Table S7: Sensitivity analysis of the HAZ models using listwise deletion method.

	Cumulative Adversities		Nurturing		Adversities*Nurturing	
**HAZ Pelotas (n=536)**	b1	p-value	b2	p-value	b3 (95% CI)	p-value
**Model 1: Base Analysis**						
Learning Opportunities	··	··	0·13 (0·03, 0·23)	0·009		
Responsive Caregiving	··	··	0·12 (0·02, 0·22)	0·022		
Cumulative Adversities: Total	-0·32 (-0·41, -0·22)	<0·001				
Cumulative Adversities: Environmental	-0·16 (-0·26, -0·07)	0·001				
Cumulative Adversities: Child	-0·48 (-0·57, -0·39)	<0·001				
**Model 2: Nurturing Adjusted for Total Adversities**						
Learning Opportunities	··	··	0·01 (-0·10, 0·12)	0·904		
Responsive Caregiving	··	··	0·04 (-0·06, 0·15)	0·417		
**Model 3: Learning Opportunities*Cumulative Adversities**						
Total	-0·32 (-0·44--0·21)	<0·001	-0·01 (-0·14-0·12)	0·859	-0·03 (-0·16, 0·09)	0·598
Environmental	-0·13 (-0·25--0·02)	0·018	0·06 (-0·07-0·19)	0·370	-0·03 (-0·15, 0·10)	0·678
Child	-0·47 (-0·56--0·37)	<0·001	0·09 (-0·00-0·18)	0·058	-0·00 (-0·09, 0·09)	0·976
**Model 4: Responsiveness*Cumulative Adversities**						
Total	-0·31 (-0·41, -0·21)	<0·001	0·05 (-0·05, 0·15)	0·349	0·04 (-0·07, 0·15)	0·435
Environmental	-0·14 (-0·24, -0·04)	0·050	0·07 (-0·04, 0·18)	0·192	0·04 (-0·07, 0·14)	0·483
Child	-0·48 (-0·57, -0·39)	<0·001	0·12 (0·03, 0·22)	0·011	0·05 (-0·05, 0·15)	0·314
**HAZ Bt20+ (n=279 to 637)**						
**Model 1: Base Analysis**						
Learning Opportunities	··	··	0·09 (0·03, 0·14)	0·002	··	··
Responsive Caregiving	··	··	0·02 (-0·04, 0·07)	0·493	··	··
Cumulative Adversities: Total	-0·14 (-0·24, -0·05)	0·004	··	··	··	··
Cumulative Adversities: Environmental	-0·09 (-0·17, -0·01)	0·023	··	··	··	··
Cumulative Adversities: Child	-0·27 (-0·34, -0·20)	<0·001	··	··	··	··
**Model 2: Nurturing Adjusted for Total Adversities**						
Learning Opportunities	··	··	0·02 (-0·08, 0·13)	0·673	··	··
Responsive Caregiving	··	··	-0·03 (-0·14, 0·07)	0·549	··	··
**Model 3: Learning Opportunities*Cumulative Adversities**						
Total	-0·14 (-0·24, -0·04)	0·007	0·03 (-0·08, 0·13)	0·630	-0·07 (-0·17, 0·04)	0·208
Environmental	-0·09 (-0·17, -0·01)	0·031	0·04 (-0·05, 0·12)	0·360	-0·08 (-0·17, 0·01)	0·080
Child	-0·26 (-0·33, -0·19)	<0·001	0·06 (-0·01, 0·13)	0·100	0·01 (-0·06, 0·08)	0·794
**Model 4: Responsiveness*Cumulative Adversities**						
Total	-0·14 (-0·24, -0·04)	0·004	-0·00 (-0·11, 0·11)	0·983	0·10 (-0·01, 0·21)	0·086
Environmental	-0·09 (-0·17, -0·01)	0·021	-0·03 (-0·11, 0·06)	0·500	0·05 (-0·04, 0·13)	0·295
Child	-0·27 (-0·34, -0·20)	<0·001	0·00 (-0·07, 0·07)	0·989	-0·01 (-0·08, 0·07)	0·862

Supplementary Table S8: Sensitivity analysis of the IQ models accounting for maternal age.[1]

	Cumulative Adversities		Nurturing		Adversities*Nurturing	
**IQ Pelotas (n=542)**	b1	p-value	b2	p-value	b3 (95% CI)	p-value
**Model 1: Base Analysis**						
Learning Opportunities	··	··	6·75 (5·54, 7·96)	<0·001	··	··
Responsive Caregiving	··	··	3·28 (1·85, 4·72)	<0·001	··	··
Cumulative Adversities: Total	-5·66 (-7·01, -4·31)	<0·001		··	··	··
Cumulative Adversities: Environmental	-5·31 (-6·51, -4·12)	<0·001		··	··	··
Cumulative Adversities: Child	-2·08 (-3·69, -0·47)	0·012		··	··	··
**Model 2: Nurturing Adjusted for Total Adversities**						
Learning Opportunities	··	··	5·19 (3·89, 6·49)	<0·001	··	··
Responsive Caregiving	··	··	2·01 (0·70, 3·33)	0·003	··	··
**Model 3: Learning Opportunities*Cumulative Adversities**						
Total	-2·80	<0·001	6·11	<0·001	1·73 (0·42, 3·04)	0·010
Environmental	-2·59	<0·001	5·77	<0·001	1·67 (0·38, 2·95)	0·011
Child	-1·33	0·086	6·71	<0·001	0·29 (-0·94, 1·52)	0·641
**Model 4: Responsiveness*Cumulative Adversities**						
Total	-5·19	<0·001	1·99	0·004	-0·15 (-1·58, 1·28)	0·836
Environmental	-4·87	<0·001	1·85	0·005	-0·12 (-1·36, 1·13)	0·852
Child	-2·03	0·014	2·98	<0·001	-0·39 (-2·44, 1·65)	0·707
**IQ Bt20+ (n=1,081)**						
**Model 1: Base Analysis**						
Learning Opportunities	··	··	1·21 (2·12, 0·31)	0·009		
Responsive Caregiving	··	··	0·63 (1·56, -0·31)	0·191		
Cumulative Adversities: Total	-2·74 (-0·92, -4·57)	0·003				
Cumulative Adversities: Environmental	-2·67 (-1·25, -4·09)	<0·001				
Cumulative Adversities: Child	-0·83 (0·35, -2·00)	0·169				
**Model 2: Nurturing Adjusted for Total Adversities**						
Learning Opportunities	··	··	0·86 (1·83, -0·11)	0·083		
Responsive Caregiving	··	··	0·65 (1·61, -0·32)	0·192		
**Model 3: Learning Opportunities*Cumulative Adversities**						
Total	-2·67 (-0·84, -4·50)	0·004	0·86 (1·84, -0·12)	0·085	-1·36 (0·62, -3·35)	0·179
Environmental	-2·60 (-1·18, -4·02)	<0·001	0·96 (1·88, 0·03)	0·043	-0·32 (1·24, -1·88)	0·686
Child	-0·72 (0·46, -1·90)	0·229	1·06 (1·99, 0·13)	0·026	0·49 (1·61, -0·62)	0·384
**Model 4: Responsiveness*Cumulative Adversities**						
Total	-2·82 (-0·99, -4·64)	0·003	1·20 (2·43, -0·03)	0·056	1·46 (3·47, -0·56)	0·156
Environmental	-2·79 (-1·36, -4·22)	<0·001	0·89 (1·90, -0·13)	0·086	1·10 (2·69, -0·50)	0·178
Child	-0·95 (0·21, -2·12)	0·109	0·75 (1·69, -0·18)	0·115	2·12 (3·35, 0·89)	0·001

[1] For the sensitivity analysis in the Pelotas dataset (weighted sample), we employed the inverse-probability-weights method accounting for differences in maternal age by using the sampling weight times the inverse propensity score weight as the weight in the final outcome analysis. For the Bt20+ dataset, linear regression models with full information maximum likelihood were adjusted for maternal age. All models were also controlled for child age.

Supplementary Table S9: Sensitivity analysis of psychosocial adjustments models accounting for maternal age.[1]

	Cumulative Adversities		Nurturing		Adversities*Nurturing	
**Psychosocial Adjustments Pelotas (n=627)**	b1	p-value	b2	p-value	b3 (95% CI)	p-value
**Model 1: Base Analysis**						
Learning Opportunities	··	··	0·11 (-0·00, 0·23)	0·052	··	··
Responsive Caregiving	··	··	-0·08 (-0·20, 0·03)	0·148	··	··
Cumulative Adversities: Total	-0·11 (-0·19, -0·03)	0·007			··	··
Cumulative Adversities: Environmental	-0·16 (-0·26, -0·06)	0·002			··	··
Cumulative Adversities: Child	0·03 (-0·11, 0·17)	0·657			··	··
**Model 2: Nurturing Adjusted for Total Adversities**						
Learning Opportunities	··	··	0·06 (-0·07, 0·19)	0·334	··	··
Responsive Caregiving	··	··	-0·13 (-0·24, -0·02)	0·026	··	··
**Model 3: Learning Opportunities*Cumulative Adversities**						
Total	-0·09	0·043	0·10	0·242	-0·03 (-0·12, 0·06)	0·530
Environmental	-0·16	0·010	0·10	0·257	-0·06 (-0·17, 0·06)	0·313
Child	0·07	0·324	0·09	0·148	0·07 (-0·06, 0·20)	0·283
**Model 4: Responsiveness*Cumulative Adversities**						
Total	-0·13	0·002	-0·18	0·070	0·02 (-0·06, 0·11)	0·583
Environmental	-0·20	<0·001	-0·15	0·129	0·00 (-0·11, 0·11)	0·992
Child	0·03	0·653	-0·11	0·072	0·11 (-0·05, 0·27)	0·163
**Psychosocial Adjustments Bt20+ (n=767)**						
**Model 1: Base Analysis**						
Learning Opportunities	··	··	0·02 (0·10, -0·06)	0·632		
Responsive Caregiving	··	··	-0·04 (0·04, -0·12)	0·343		
Cumulative Adversities: Total	-0·08 (0·09, -0·25)	0·336				
Cumulative Adversities: Environmental	0·03 (0·16, -0·10)	0·651				
Cumulative Adversities: Child	0·02 (0·13, -0·10)	0·770				
**Model 2: Nurturing Adjusted for Total Adversities**						
Learning Opportunities	··	··	0·01 (0·10, -0·08)	0·873		
Responsive Caregiving	··	··	-0·04 (0·04, -0·13)	0·296		
**Model 3: Learning Opportunities*Cumulative Adversities**						
Total	-0·09 (0·08, -0·26)	0·305	-0·01 (0·08, -0·10)	0·848	0·12 (0·30, -0·05)	0·159
Environmental	0·03 (0·16, -0·10)	0·629	0·03 (0·11, -0·06)	0·546	-0·02 (0·12, -0·15)	0·811
Child	0·03 (0·15, -0·08)	0·591	-0·01 (0·08, -0·10)	0·852	0·10 (0·21, -0·01)	0·077
**Model 4: Responsiveness*Cumulative Adversities**						
Total	-0·09 (0·08, -0·25)	0·309	-0·08 (0·01, -0·18)	0·084	-0·17 (0·01, -0·35)	0·063
Environmental	0·04 (0·16, -0·09)	0·558	-0·05 (0·03, -0·13)	0·235	-0·12 (0·01, -0·26)	0·073
Child	0·02 (0·13, -0·10)	0·754	-0·04 (0·04, -0·12)	0·339	0·00 (0·11, -0·10)	0·936

[1] For the sensitivity analysis in the Pelotas dataset (weighted sample), we employed the inverse-probability-weights method accounting for differences in maternal age by using the sampling weight times the inverse propensity score weight as the weight in the final outcome analysis. For the Bt20+ dataset, linear regression models with full information maximum likelihood were adjusted for maternal age. All models were also controlled for child age.

Supplementary Table S10: Sensitivity analysis of the HAZ models accounting for maternal age.[1]

	Cumulative Adversities		Nurturing		Adversities*Nurturing	
**HAZ Pelotas (n=536)**	b1	p-value	b2	p-value	b3 (95% CI)	p-value
**Model 1: Base Analysis**						
Learning Opportunities	··	··	0·15 (0·04, 0·26)	0·008		
Responsive Caregiving	··	··	0·09 (-0·01, 0·20)	0·081		
Cumulative Adversities: Total	-0·18 (-0·25, -0·11)	<0·001				
Cumulative Adversities: Environmental	-0·12 (-0·22, -0·03)	0·012				
Cumulative Adversities: Child	-0·60 (-0·73, -0·48)	<0·001				
**Model 2: Nurturing Adjusted for Total Adversities**						
Learning Opportunities	··	··	0·05 (-0·07, 0·17)	0·458		
Responsive Caregiving	··	··	0·03 (-0·08, 0·13)	0·643		
**Model 3: Learning Opportunities*Cumulative Adversities**						
Total	-0·17	<0·001	0·07	0·387	-0·02 (-0·12, 0·07)	0·614
Environmental	-0·08	0·128	0·13	0·134	-0·03 (-0·15, 0·09)	0·670
Child	-0·59	<0·001	0·12	0·056	-0·01 (-0·12, 0·11)	0·932
**Model 4: Responsiveness*Cumulative Adversities**						
Total	-0·18	<0·001	-0·02	0·827	0·03 (-0·05, 0·11)	0·516
Environmental	-0·11	0·031	0·00	0·989	0·03 (-0·07, 0·14)	0·534
Child	-0·60	<0·001	0·07	0·229	0·07 (-0·06, 0·20)	0·308
**HAZ Bt20+ (n=1,130)**						
**Model 1: Base Analysis**						
Learning Opportunities	··	··	0·08 (0·14, 0·03)	0·003		
Responsive Caregiving	··	··	0·02 (0·08, -0·03)	0·447		
Cumulative Adversities: Total	-0·13 (-0·04, -0·23)	0·008				
Cumulative Adversities: Environmental	-0·09 (-0·00, -0·17)	0·047				
Cumulative Adversities: Child	-0·25 (-0·19, -0·32)	<0.001				
**Model 2: Nurturing Adjusted for Total Adversities**						
Learning Opportunities	··	··	0·06 (0·12, 0·00)	0·036		
Responsive Caregiving	··	··	0·02 (0·08, -0·04)	0·481		
**Model 3: Learning Opportunities*Cumulative Adversities**						
Total	-0·13 (-0·03, -0·23)	0·010	0·06 (0·12, 0·01)	0·029	-0·06 (0·05, -0·16)	0·298
Environmental	-0·08 (0·00, -0·16)	0·057	0·08 (0·13, 0·02)	0·007	-0·08 (0·01, -0·17)	0·087
Child	-0·25 (-0·18, -0·31)	<0.001	0·06 (0·11, 0·00)	0·041	0·01 (0·08, -0·06)	0·770
**Model 4: Responsiveness*Cumulative Adversities**						
Total	-0·14 (-0·04, -0·23)	0·007	0·05 (0·12, -0·02)	0·151	0·09 (0·20, -0·02)	0·123
Environmental	-0·09 (-0·00, -0·17)	0·043	0·03 (0·09, -0·03)	0·284	0·04 (0·13, -0·05)	0·375
Child	-0·25 (-0·19, -0·32)	<0.001	0·02 (0·07, -0·04)	0·529	-0·00 (0·07, -0·07)	0·916

[1] For the sensitivity analysis in the Pelotas dataset (weighted sample), we employed the inverse-probability-weights method accounting for differences in maternal age by using the sampling weight times the inverse propensity score weight as the weight in the final outcome analysis. For the Bt20+ dataset, linear regression models with full information maximum likelihood were adjusted for maternal age. All models were also controlled for child age.
